# 4-Bromo-*N* ^2^,*N* ^2^,*N* ^6^,*N* ^6^-tetra­ethyl­pyridine-2,6-dicarboxamide

**DOI:** 10.1107/S1600536810028837

**Published:** 2010-07-24

**Authors:** Daniel T. de Lill, Ana de Bettencourt-Dias

**Affiliations:** a University of Nevada, Reno, Department of Chemistry, 1664 N. Virginia St, Reno, NV 89557-0216, USA

## Abstract

The title compound, C_15_H_22_BrN_3_O_2_, consists of a pyridine ring with a bromine atom in the *para* position and two diethyl­amide groups in the *ortho* positions of the ring. Despite the positions of the three substituents on the pyridine ring, the mol­ecule does not exhibit either local or crystallographic twofold symmetry as the two diethyl­amido units exhibit significantly different N_py_—C—C—N_am_ torsion angles of 46.3 (4) and 62.7 (4)° (py is pyridine and am is amine). Inter­molecular C—H⋯O inter­actions support the packing.

## Related literature

The title compound has been investigated as a sensitizer of lanthanide ion luminescence. For uses of this ligand and its derivatives, see: de Bettencourt-Dias *et al.* (2006 ▶); Renaud *et al.* (1997 ▶). For other structures involving this moiety, see: Muller *et al.* (2003 ▶).
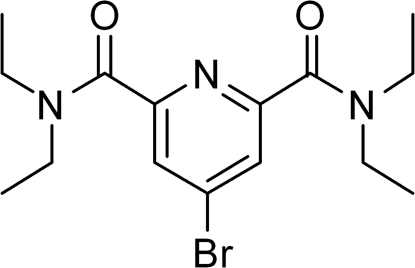


## Experimental

### 

#### Crystal data

C_15_H_22_BrN_3_O_2_
*M* *_r_* = 356.27
Orthorhombic, 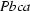

*a* = 17.7096 (4) Å
*b* = 8.4987 (2) Å
*c* = 21.5013 (4) Å
*V* = 3236.13 (12) Å^3^
*Z* = 8
Mo *K*α radiation
μ = 2.55 mm^−1^
*T* = 100 K
0.17 × 0.08 × 0.07 mm

#### Data collection

Bruker APEX CCD diffractometer
Absorption correction: multi-scan (*SADABS*; Sheldrick, 2004 ▶) *T*_min_ = 0.670, *T*_max_ = 0.852
23172 measured reflections
3392 independent reflections
2499 reflections with *I* > 2σ(*I*)
*R*_int_ = 0.045

#### Refinement

*R*[*F* ^2^ > 2σ(*F* ^2^)] = 0.039
*wR*(*F* ^2^) = 0.101
*S* = 1.05
3392 reflections
194 parameters
H-atom parameters constrained
Δρ_max_ = 0.56 e Å^−3^
Δρ_min_ = −0.58 e Å^−3^

### 

Data collection: *SMART* (Bruker, 2001 ▶); cell refinement: *SAINT* (Bruker, 2001 ▶); data reduction: *SAINT*; program(s) used to solve structure: *SHELXS97* (Sheldrick, 2008 ▶); program(s) used to refine structure: *SHELXL97* (Sheldrick, 2008 ▶); molecular graphics: *DIAMOND* (Brandenburg, 1999 ▶); software used to prepare material for publication: *SHELXTL* (Sheldrick, 2008 ▶).

## Supplementary Material

Crystal structure: contains datablocks I, global. DOI: 10.1107/S1600536810028837/fl2308sup1.cif

Structure factors: contains datablocks I. DOI: 10.1107/S1600536810028837/fl2308Isup2.hkl

Additional supplementary materials:  crystallographic information; 3D view; checkCIF report

## Figures and Tables

**Table 1:** Hydrogen-bond geometry (Å, °)

*D*—H⋯*A*	*D*—H	H⋯*A*	*D*⋯*A*	*D*—H⋯*A*
C2—H2⋯O1^i^	0.95	2.54	3.429 (4)	156
C8—H8*A*⋯O2^ii^	0.99	2.59	3.250 (4)	124
C9—H9*A*⋯O1^iii^	0.98	2.48	3.447 (4)	168
C9—H9*B*⋯O2^ii^	0.98	2.87	3.479 (4)	121
C10—H10*A*⋯O1^iii^	0.99	2.72	3.652 (4)	157
C13—H13*A*⋯O2^iv^	0.99	2.63	3.415 (4)	135
C14—H14*A*⋯O2^iv^	0.99	2.73	3.444 (4)	130
C15—H15*A*⋯O2^iv^	0.98	2.99	3.525 (4)	115

Symmetry codes: (i) 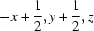; (ii) 
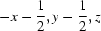
; (iii) 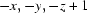; (iv) 
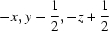
.

**Fig. 1.**
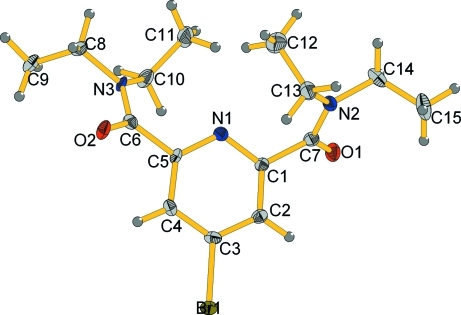
Molecular structure of the title compound, with atom numbering (except H atoms) and 50% probability displacement ellipsoids for non-H atoms.

**Fig. 2.**
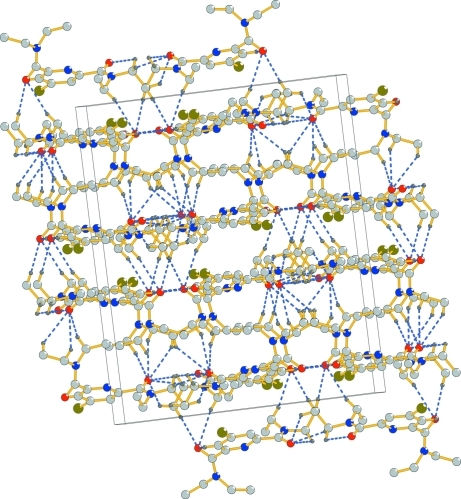
Packing diagram of the title compound viewed down the b axis. The hydrogen bonds are drawn as dashed lines. Hydrogen atoms not involved in hydrogen bonding interactions were omitted for clarity.

## supplementary crystallographic information

### Comment

The title compound, shown in Figure 1 and Scheme 1, is often utillized as the chelating and sensitizing moiety in ligands for lanthanide ion luminescent complexes, such as in (*tert*-butoxycarbonyl) alanine methyl ester, the structure of which has been reported (Muller *et al.*, 2003) or in a *N*,*N*,*N*',*N*'-tetraethylpyridine-2,6,dicarboxamide-based ligand (Renaud *et al.*, 1997). It has also been used as an intermediate to ligands capable of coordinating lanthanide ions (de Bettencourt-Dias *et al.*, 2006) and as such was isolated in our research group. It consists of a pyridine ring with a bromide in position 4 and diethylamide groups in positions 2 and 5. This molecule is devoid of crystallographic symmetry and the asymmetric unit comprises one molecule. While at first impression the amide groups seem to be related by a twofold axis, closer inspection shows that they are different. This is evidenced by the torsion angles between the groups and the pyridine ring. The torsion angle for the atoms N1—C1—C7—N2 is 62.7 (4)° and the torsion angle for N1—C5—C6—N3 is 46.3 (4)°. The observed difference between the two ethyl groups might be a consequence of the C—H···O hydrogen bond interactions in which they are involved and which help support the packing structure, shown in Figure 2. Despite the presence of the pyridine rings and of the bromine atoms, π–π, C—Br···π or C—H···Br interactions are not observed.

### Experimental

The title compound was synthesized as follows. PBr_5_ was obtained by slowly adding 8 ml of PBr_3_ to 3.5 ml of Br_2_ in 20 ml hexanes at 0 °C. After one hour, the hexanes were decanted and 5 g of chelidamic acid were added. The mixture was heated for six hours (85–90 °C.) The acid bromide which formed in this step was then extracted with chloroform and used without further purification in the next step.

The solution of the acid bromide in CHCl_3_ was slowly added to 2-chlorodiethylamine (2 eq.) in 50 ml H_2_O/KOH (5 eq.) at 0 °C, stirred for 20 minutes, then allowed to slowly warm to room temperature. The aqueous layer was washed and discarded. The organic layer was concentrated, filtered, and the product rinsed with water and hexanes. Crystals were grown by allowing a solution of (I) in CHCl_3_ to evaporate overnight. Overall yield: 5.7 g, 59%. 1H NMR (400 MHz, CDCl_3_) p.p.m.: 7.80 (m, 2H), 3.55 (q, 4H), 3.33 (q, 4H), 1.25 (t, 6H), 1.15 (dd, 6H).

### Refinement

Hydrogen atoms were positioned geometrically using a riding model with C—H = 0.95, 0.99 and 0.98 Å for aromatic CH and aliphatic CH_2_ and CH_3_ H atoms, respectively, and *U*_iso_(H)=1.2–1.5 *U*_eq_(C).

### Crystal data

**Table:**

C_15_H_22_BrN_3_O_2_	*F*(000) = 1472
*M**_r_* = 356.27	*D*_x_ = 1.462 Mg m^−^^3^
Orthorhombic, *P**b**c**a*	Mo *K*α radiation, λ = 0.71073 Å
Hall symbol: -P 2ac 2ab	Cell parameters from 4936 reflections
*a* = 17.7096 (4) Å	θ = 2.8–27.8°
*b* = 8.4987 (2) Å	µ = 2.55 mm^−^^1^
*c* = 21.5013 (4) Å	*T* = 100 K
*V* = 3236.13 (12) Å^3^	Needle, clear light yellow
*Z* = 8	0.17 × 0.08 × 0.07 mm

### Data collection

**Table:**

Bruker APEX CCD diffractometer	3392 independent reflections
Radiation source: fine-focus sealed tube	2499 reflections with *I* > 2σ(*I*)
graphite	*R*_int_ = 0.045
phi and ω scans	θ_max_ = 26.7°, θ_min_ = 1.9°
Absorption correction: multi-scan (*SADABS*; Sheldrick, 2004)	*h* = −22→16
*T*_min_ = 0.670, *T*_max_ = 0.852	*k* = −10→10
23172 measured reflections	*l* = −26→27

### Refinement

**Table:**

Refinement on *F*^2^	Primary atom site location: structure-invariant direct methods
Least-squares matrix: full	Secondary atom site location: difference Fourier map
*R*[*F*^2^ > 2σ(*F*^2^)] = 0.039	Hydrogen site location: inferred from neighbouring sites
*wR*(*F*^2^) = 0.101	H-atom parameters constrained
*S* = 1.05	*w* = 1/[σ^2^(*F*_o_^2^) + (0.0375*P*)^2^ + 6.2825*P*] where *P* = (*F*_o_^2^ + 2*F*_c_^2^)/3
3392 reflections	(Δ/σ)_max_ = 0.001
194 parameters	Δρ_max_ = 0.56 e Å^−^^3^
0 restraints	Δρ_min_ = −0.58 e Å^−^^3^

### Special details

**Table:**

Geometry. All e.s.d.'s (except the e.s.d. in the dihedral angle between two l.s. planes) are estimated using the full covariance matrix. The cell e.s.d.'s are taken into account individually in the estimation of e.s.d.'s in distances, angles and torsion angles; correlations between e.s.d.'s in cell parameters are only used when they are defined by crystal symmetry. An approximate (isotropic) treatment of cell e.s.d.'s is used for estimating e.s.d.'s involving l.s. planes.
Refinement. Refinement of *F*^2^ against ALL reflections. The weighted *R*-factor *wR* and goodness of fit *S* are based on *F*^2^, conventional *R*-factors *R* are based on *F*, with *F* set to zero for negative *F*^2^. The threshold expression of *F*^2^ > σ(*F*^2^) is used only for calculating *R*-factors(gt) *etc*. and is not relevant to the choice of reflections for refinement. *R*-factors based on *F*^2^ are statistically about twice as large as those based on *F*, and *R*- factors based on ALL data will be even larger.

### Fractional atomic coordinates and isotropic or equivalent isotropic displacement parameters (Å^2^)

**Table:**

	*x*	*y*	*z*	*U*_iso_*/*U*_eq_	
Br1	0.122569 (19)	0.65446 (4)	0.436078 (17)	0.02253 (12)	
N1	0.02418 (15)	0.1655 (3)	0.38110 (12)	0.0146 (6)	
C2	0.13042 (19)	0.3352 (4)	0.39707 (14)	0.0152 (7)	
H2	0.1835	0.3507	0.3959	0.018*	
C6	−0.10501 (18)	0.2584 (4)	0.39805 (14)	0.0157 (7)	
C3	0.08262 (19)	0.4540 (4)	0.41694 (15)	0.0166 (7)	
C4	0.00592 (19)	0.4288 (4)	0.41939 (14)	0.0164 (7)	
H4	−0.0277	0.5091	0.4327	0.020*	
C5	−0.02047 (17)	0.2821 (4)	0.40168 (14)	0.0126 (6)	
C1	0.09906 (18)	0.1947 (4)	0.37915 (14)	0.0133 (7)	
C7	0.14800 (18)	0.0595 (4)	0.35846 (15)	0.0165 (7)	
C9	−0.26126 (18)	0.1403 (4)	0.46580 (16)	0.0214 (8)	
H9A	−0.2419	0.0830	0.5020	0.032*	
H9B	−0.3144	0.1130	0.4592	0.032*	
H9C	−0.2568	0.2538	0.4731	0.032*	
C15	0.2606 (2)	−0.0792 (5)	0.25542 (17)	0.0318 (10)	
H15A	0.2525	−0.0110	0.2193	0.048*	
H15B	0.2899	−0.1718	0.2428	0.048*	
H15C	0.2885	−0.0212	0.2874	0.048*	
C14	0.1854 (2)	−0.1311 (4)	0.28117 (17)	0.0261 (8)	
H14A	0.1579	−0.1914	0.2490	0.031*	
H14B	0.1939	−0.2017	0.3171	0.031*	
C13	0.0897 (2)	0.0736 (4)	0.25344 (16)	0.0226 (8)	
H13A	0.1166	0.0742	0.2131	0.027*	
H13B	0.0794	0.1843	0.2650	0.027*	
C8	−0.21630 (18)	0.0963 (4)	0.40922 (16)	0.0194 (7)	
H8A	−0.2251	−0.0161	0.3996	0.023*	
H8B	−0.2343	0.1591	0.3734	0.023*	
C11	−0.0732 (2)	−0.1413 (4)	0.4195 (2)	0.0338 (10)	
H11A	−0.1167	−0.1810	0.3961	0.051*	
H11B	−0.0557	−0.2222	0.4486	0.051*	
H11C	−0.0324	−0.1150	0.3905	0.051*	
C10	−0.0960 (2)	0.0050 (4)	0.45545 (17)	0.0218 (8)	
H10A	−0.1294	−0.0264	0.4902	0.026*	
H10B	−0.0502	0.0534	0.4736	0.026*	
C12	0.0153 (2)	−0.0114 (5)	0.24541 (19)	0.0371 (10)	
H12A	0.0249	−0.1224	0.2358	0.056*	
H12B	−0.0131	0.0368	0.2113	0.056*	
H12C	−0.0140	−0.0037	0.2839	0.056*	
N3	−0.13501 (14)	0.1225 (3)	0.41736 (13)	0.0139 (6)	
N2	0.13906 (15)	0.0027 (3)	0.30094 (12)	0.0172 (6)	
O2	−0.14317 (12)	0.3659 (3)	0.37517 (11)	0.0202 (5)	
O1	0.19438 (13)	0.0070 (3)	0.39535 (10)	0.0209 (5)	

### Atomic displacement parameters (Å^2^)

**Table:**

	*U*^11^	*U*^22^	*U*^33^	*U*^12^	*U*^13^	*U*^23^
Br1	0.0197 (2)	0.01536 (18)	0.0325 (2)	−0.00308 (14)	0.00063 (16)	−0.00522 (15)
N1	0.0166 (14)	0.0131 (13)	0.0141 (13)	−0.0011 (11)	0.0015 (11)	0.0005 (11)
C2	0.0151 (17)	0.0178 (16)	0.0127 (15)	−0.0028 (14)	−0.0021 (13)	0.0008 (13)
C6	0.0174 (17)	0.0162 (16)	0.0135 (16)	0.0016 (14)	0.0000 (14)	−0.0022 (13)
C3	0.0200 (18)	0.0113 (16)	0.0185 (17)	−0.0017 (14)	−0.0041 (14)	−0.0008 (13)
C4	0.0178 (18)	0.0156 (16)	0.0157 (16)	0.0031 (14)	0.0034 (14)	−0.0007 (13)
C5	0.0079 (15)	0.0167 (16)	0.0131 (16)	0.0002 (13)	−0.0003 (13)	0.0027 (13)
C1	0.0127 (16)	0.0161 (16)	0.0111 (15)	−0.0004 (13)	−0.0018 (13)	−0.0003 (12)
C7	0.0169 (17)	0.0138 (16)	0.0186 (17)	−0.0028 (13)	0.0022 (14)	0.0010 (13)
C9	0.0112 (18)	0.0279 (19)	0.0252 (18)	−0.0027 (14)	−0.0031 (15)	0.0034 (16)
C15	0.033 (2)	0.037 (2)	0.026 (2)	0.0149 (19)	0.0127 (17)	0.0035 (17)
C14	0.036 (2)	0.0167 (18)	0.0251 (19)	0.0075 (16)	0.0013 (17)	−0.0057 (15)
C13	0.020 (2)	0.028 (2)	0.0197 (18)	0.0047 (16)	−0.0041 (15)	−0.0047 (15)
C8	0.0145 (17)	0.0184 (16)	0.0253 (18)	−0.0045 (14)	−0.0016 (15)	0.0007 (15)
C11	0.022 (2)	0.0176 (18)	0.062 (3)	0.0013 (15)	0.013 (2)	0.0019 (18)
C10	0.0134 (17)	0.0191 (17)	0.033 (2)	0.0004 (14)	−0.0016 (15)	0.0082 (15)
C12	0.026 (2)	0.055 (3)	0.030 (2)	−0.003 (2)	−0.0050 (18)	−0.016 (2)
N3	0.0051 (13)	0.0152 (14)	0.0213 (14)	−0.0007 (10)	0.0014 (11)	0.0023 (11)
N2	0.0154 (15)	0.0169 (14)	0.0192 (15)	0.0034 (11)	−0.0015 (12)	−0.0030 (12)
O2	0.0104 (11)	0.0212 (13)	0.0291 (13)	0.0022 (9)	0.0010 (10)	0.0068 (10)
O1	0.0163 (12)	0.0237 (13)	0.0228 (13)	0.0049 (10)	−0.0011 (11)	−0.0005 (10)

### Geometric parameters (Å, °)

**Table:**

Br1—C3	1.890 (3)	C15—H15C	0.9800
N1—C5	1.343 (4)	C14—N2	1.465 (4)
N1—C1	1.350 (4)	C14—H14A	0.9900
C2—C1	1.372 (4)	C14—H14B	0.9900
C2—C3	1.385 (4)	C13—N2	1.474 (4)
C2—H2	0.9500	C13—C12	1.512 (5)
C6—O2	1.238 (4)	C13—H13A	0.9900
C6—N3	1.338 (4)	C13—H13B	0.9900
C6—C5	1.513 (4)	C8—N3	1.467 (4)
C3—C4	1.376 (5)	C8—H8A	0.9900
C4—C5	1.385 (4)	C8—H8B	0.9900
C4—H4	0.9500	C11—C10	1.519 (5)
C1—C7	1.506 (4)	C11—H11A	0.9800
C7—O1	1.226 (4)	C11—H11B	0.9800
C7—N2	1.337 (4)	C11—H11C	0.9800
C9—C8	1.501 (5)	C10—N3	1.465 (4)
C9—H9A	0.9800	C10—H10A	0.9900
C9—H9B	0.9800	C10—H10B	0.9900
C9—H9C	0.9800	C12—H12A	0.9800
C15—C14	1.509 (5)	C12—H12B	0.9800
C15—H15A	0.9800	C12—H12C	0.9800
C15—H15B	0.9800		
			
C5—N1—C1	116.9 (3)	C15—C14—H14B	109.2
C1—C2—C3	118.2 (3)	H14A—C14—H14B	107.9
C1—C2—H2	120.9	N2—C13—C12	113.6 (3)
C3—C2—H2	120.9	N2—C13—H13A	108.8
O2—C6—N3	122.9 (3)	C12—C13—H13A	108.8
O2—C6—C5	117.6 (3)	N2—C13—H13B	108.8
N3—C6—C5	119.5 (3)	C12—C13—H13B	108.8
C4—C3—C2	120.2 (3)	H13A—C13—H13B	107.7
C4—C3—Br1	120.1 (2)	N3—C8—C9	112.7 (3)
C2—C3—Br1	119.7 (2)	N3—C8—H8A	109.1
C3—C4—C5	117.5 (3)	C9—C8—H8A	109.1
C3—C4—H4	121.2	N3—C8—H8B	109.1
C5—C4—H4	121.2	C9—C8—H8B	109.1
N1—C5—C4	123.8 (3)	H8A—C8—H8B	107.8
N1—C5—C6	117.9 (3)	C10—C11—H11A	109.5
C4—C5—C6	117.9 (3)	C10—C11—H11B	109.5
N1—C1—C2	123.3 (3)	H11A—C11—H11B	109.5
N1—C1—C7	115.7 (3)	C10—C11—H11C	109.5
C2—C1—C7	120.9 (3)	H11A—C11—H11C	109.5
O1—C7—N2	123.1 (3)	H11B—C11—H11C	109.5
O1—C7—C1	118.2 (3)	N3—C10—C11	113.5 (3)
N2—C7—C1	118.7 (3)	N3—C10—H10A	108.9
C8—C9—H9A	109.5	C11—C10—H10A	108.9
C8—C9—H9B	109.5	N3—C10—H10B	108.9
H9A—C9—H9B	109.5	C11—C10—H10B	108.9
C8—C9—H9C	109.5	H10A—C10—H10B	107.7
H9A—C9—H9C	109.5	C13—C12—H12A	109.5
H9B—C9—H9C	109.5	C13—C12—H12B	109.5
C14—C15—H15A	109.5	H12A—C12—H12B	109.5
C14—C15—H15B	109.5	C13—C12—H12C	109.5
H15A—C15—H15B	109.5	H12A—C12—H12C	109.5
C14—C15—H15C	109.5	H12B—C12—H12C	109.5
H15A—C15—H15C	109.5	C6—N3—C8	118.9 (3)
H15B—C15—H15C	109.5	C6—N3—C10	125.1 (3)
N2—C14—C15	112.0 (3)	C8—N3—C10	115.2 (3)
N2—C14—H14A	109.2	C7—N2—C14	118.8 (3)
C15—C14—H14A	109.2	C7—N2—C13	124.3 (3)
N2—C14—H14B	109.2	C14—N2—C13	116.7 (3)

### Hydrogen-bond geometry (Å, °)

**Table:**

*D*—H···*A*	*D*—H	H···*A*	*D*···*A*	*D*—H···*A*
C2—H2···O1^i^	0.95	2.54	3.429 (4)	156
C8—H8A···O2^ii^	0.99	2.59	3.250 (4)	124
C8—H8B···O2	0.99	2.39	2.732 (4)	100
C9—H9A···O1^iii^	0.98	2.48	3.447 (4)	168
C9—H9B···O2^ii^	0.98	2.87	3.479 (4)	121
C10—H10A···O1^iii^	0.99	2.72	3.652 (4)	157
C13—H13A···O2^iv^	0.99	2.63	3.415 (4)	135
C14—H14A···O2^iv^	0.99	2.73	3.444 (4)	130
C15—H15A···O2^iv^	0.98	2.99	3.525 (4)	115

Symmetry codes: (i) −*x*+1/2, *y*+1/2, *z*; (ii) −*x*−1/2, *y*−1/2, *z*; (iii) −*x*, −*y*, −*z*+1; (iv) −*x*, *y*−1/2, −*z*+1/2.
